# Evaluating the Efficacy of Immunotherapy in Fragile Hospitalized Patients

**DOI:** 10.3390/curroncol31110518

**Published:** 2024-11-10

**Authors:** Charles Vincent Rajadurai, Guillaume Gagnon, Catherine Allard, Mandy Malick, Michel Pavic

**Affiliations:** 1 Department of Medical Oncology, McGill University Health Centre (MUHC), McGill University, Montreal, QC H4A-3J1, Canada; 2 Department of Hemato-Oncology, Centre Hospitalier Universitaire de Sherbrooke (CHUS), Sherbrooke University, Sherbrooke, QC J1H-5N4, Canada; guillaume.gagnon6@usherbrooke.ca (G.G.); pro-recherche-med@usherbrooke.ca (M.M.); michel.pavic@usherbrooke.ca (M.P.); 3 Centre de Recherche du Centre Hospitalier Universitaire de Sherbrooke (CHUS), Sherbrooke, QC J1H-5N4, Canada; catherine.allard.ciussse-chus@ssss.gouv.qc.ca; 4 Department of Medicine, Centre Hospitalier Université de Sherbrooke (CHUS), Sherbrooke University, Sherbrooke, QC J1H-5N4, Canada; 5 Institut de Recherche sur le Cancer de l’Université de Sherbrooke (IRCUS), Sherbrooke University—Health Campus, Sherbrooke, QC J1E 4K8, Canada

**Keywords:** immunotherapy, adverse events, fragile patients, hospitalized patient, effectiveness of immune checkpoint inhibition

## Abstract

Background: Immunotherapy is the cornerstone of treatment for many cancers. The effectiveness of immunotherapy in hospitalized patients is unknown due to the exclusion of this fragile population from clinical trials. This study evaluates the efficacy of immunotherapy in fragile hospitalized patients. Method: We conducted a single-center retrospective study involving 49 patients who started an immunotherapy (IO) during a hospitalization or within 3 months after a hospitalization at the Centre Hospitalier de l’Université de Sherbrooke (CHUS). Efficacy analysis included objective response rate (ORR), overall survival (OS), and progression-free survival (PFS). Results: Immunotherapy resulted in 30.6% of all grades combined and 18.4% of grade three to four immune-related adverse events (irAE). Efficacy outcomes were inferior in the fragile cohort of patients with ORR of 38.9%, PFS of 2.8 months (95% CI [2.17–3.35]), and OS of 3.2 months (95% CI [1.60–4.84]). Performance status of ECOG three to four compared to ECOG zero predicts poor OS (HR 5.666 [1.207–26.594]; *p* = 0.028) and PFS (HR 4.136 [0.867–19.733]; *p* = 0.075). Fitness to receive four to six cycles (HR 0.335 [0.152–0.0.738]; *p* < 0.007) or more predicts greater OS compared to one to three cycles of immunotherapy. Low levels of serum albumin (HR 0.917 [0.852–0.987]; *p* = 0.021) and elevated levels of serum LDH (HR 2.224 [1.469–3.367]; *p* < 0.001) are associated with a reduced OS. Conclusion: The effectiveness of immunotherapy in fragile hospitalized patients is compromised, although they exhibit significant irAE. Excellent performance status, fitness to receive many IO treatments, and normal levels of serum LDH and albumin may be useful in selecting patients who will benefit from immunotherapy.

## 1. Introduction

About 20 million new cancer cases are diagnosed worldwide each year [1]. Cancer is the second leading cause of mortality, accounting for approximately 10 million cancer-related deaths annually [2]. As a result, cancer diagnosis and treatment have a tremendous impact on health economics. International collaborative efforts have been ongoing for more than half a century to identify effective treatment against cancer. One of the most exciting outcomes from these efforts in the recent past is the development of immunotherapy, particularly “immune checkpoint blockade” (ICB) [3]. It has extended the therapeutic arsenal against cancer and radically changed the landscape of cancer treatment [2]. Since the FDA approved the first immune checkpoint inhibitor (ICI), Ipilimumab, in 2011, numerous other ICIs have flooded the market and received FDA approval for treatment indications for many cancers in the metastatic and perioperative setting [4,5]. The ICB relies on a functional host immune system to manipulate T-lymphocytes to target cancer cells. Specifically, the immune checkpoints mediated by cytotoxic T-lymphocyte associated antigen-4 (CTLA-4), programmed cell death-1 (PD1), and programmed cell death ligand 1 (PD-L1) inhibit T-cell activation through their interaction with antigen-presenting cells or tumor cells [6]. Therefore, the blockade of these immune checkpoints with monoclonal antibodies (ICI) in an immune-competent host suppresses the inhibitory pathways of the T-lymphocytes against cancer cells, which in turn enhances its antitumor activity [6,7]. To this effect, in some cancer types, including melanoma, unprecedented sustained long-term response has been documented [8].

As treatment regimens containing ICI in the form of combination immunotherapies, immunotherapy-chemotherapy, or immunotherapy-tyrosine kinase inhibitor (TKI) continue to multiply, a vast array of irAEs are also becoming evident [9]. These irAEs are unpredictable and diverse in presentation and severity. Adverse effects like thyroiditis, dermatitis, arthritis, and colitis are common [9,10]. Although some irAEs like myocarditis, hepatitis, and pneumonitis are rare, they are potentially life threatening [10]. Adverse events of this nature are caused by an uncontrolled autoimmunity against healthy tissues [11]. It is thought that frail patients are unfit to tolerate the severe side effects of chemotherapy, but their fitness to receive immunotherapy is unknown given the unpredictable nature of irAE, in terms of the spectrum and the severity [12,13]. For this reason, even though chemotherapy is not acceptable, ICI therapy is often deemed acceptable in frail patients [9,14,15]. Also, biomarker-matched ICI treatment offers hope for sustained long-term response [16,17]. As ICIs rely entirely on the functional host immune system, their efficacy in frail hospitalized patients who may have a weakened immune system is currently unknown [18]. Unfortunately, the frail population was excluded from the phase three randomized controlled trials. As such, real-world evidence is needed to fill this gap in knowledge regarding the efficacy and safety of ICIs in frail hospitalized patients. To this effect, a recent retrospective study reported that 46% of patients who had received at least one dose of an anti-PD-1 or an anti-PD-L1 while being hospitalized died in the same hospital stay or were discharged to hospice [19]. Another multicenter retrospective study reported that 44.7% of patients treated with ICIs in inpatient settings did not have FDA-approved indications at the time of administration [20]. Moreover, several studies have reported that patients exhibit signs of concomitant immune suppression and hyperinflammation to varying degree immediately following their hospital stay. While many hypotheses are put forward to explain this phenomenon, infectious complications are widely accepted as a major reason, and could predispose to concomitant immune suppression and hyperinflammation lasting up to a year after hospitalization [21,22,23]. Taken together, it raises the questions about the role of ICIs in fragile hospitalized cancer patients.

In this regard, the primary goal of our study is to examine the efficacy and safety of ICIs on hospitalized patients or patients who have been hospitalized 3 months prior to receiving their immunotherapy. As noted above, these patients are considered fragile with a high likelihood of impaired immunity, regardless of performance status or biologic and anthropometrics parameters. This work will shed light not only on the efficacy and safety profile of ICIs on fragile patients but also identify key variables that may be helpful in selecting candidates to receive ICIs, even if they are deemed fragile due to a current or recent hospitalization.

## 2. Material and Methods

### 2.1. Study Design

We conducted a non-experimental descriptive and retrospective study at the Centre Hospitalier de l’Université de Sherbrooke (CHUS) from January 2018 to December 2021. The approval for this study was obtained from the Research Ethics Board of the CHUS on 10 January 2022 and renewed on 10 January 2023 (Project Approval Number 2022–4497).

### 2.2. Patient Enrollment

We enrolled patients aged 18 years and above with various types of cancer for whom immunotherapy with a checkpoint inhibitor was first initiated during or up to 3 months after their hospital stay from January 2018 to December 2021. We chose to include patients up to 3 months after hospitalization in order to increase statistical power while ensuring to select fragile patients with a high likelihood of impaired immunity at the start of immunotherapy treatment. Patients were excluded if they had received concomitant chemotherapy or if they were in a double-blinded study where it would not be possible to know if they received an ICI as part of their cancer treatment.

### 2.3. Data Collection

Utilizing the local research platforms DCI-Ariane and CIRÉSS (Centre Informatisé de Recherche Évaluative en Services et Soins de Santé), and after receiving approval from the Direction des Services Professionnels (DSP) du CHUS, we established a comprehensive database. This database was constructed through a systematic probabilistic sampling methodology, encompassing all patients who fulfilled our predetermined inclusion criteria. Patient data were meticulously extracted from their electronic medical records.

### 2.4. Outcome Measures

The primary endpoints were progression-free survival (PFS) and overall survival (OS) to evaluate the efficacy of ICIs in this population. If the cancer progressed during ICI treatment, PFS was measured as the time between the first immunotherapy treatment and the first radiological evidence of progression. Radiological evidence of progression had to be noted on two consecutive scans (MRI, CT scan, or PET scan) to rule out pseudo-progression. Radiological progression was determined by the iRECIST guideline [24] if mentioned in the radiologist report or by the clinical judgment of the radiologist and oncologist otherwise.

If there was a response to treatment or a stable disease, PFS was censured at the time between the first ICI treatment and the last scan. If a patient received the first ICI treatment shortly before the start of data collection (1 March 2022), a following scan could not be performed, and these patients were therefore excluded from the PFS analysis. OS was defined as the time between the first cycle of ICIs and patient death if the patient was deceased, and if the patient was still alive, censured at the time between the first cycle of ICIs and the last contact date in the medical record.

The secondary endpoints were to assess specific patient, biochemical, disease, and treatment characteristics to identify the vulnerable population that would least benefit from ICI treatment. For the patient characteristics, the demographic and anthropometric data were collected. The performance status at the initiation of ICB was graded from zero to four in accordance with the Eastern Cooperative Oncology Group (ECOG) noted at the time of ICI initiation by the oncologist [25]. The type of immunotherapy as well as the number of cycles received and the line of treatment was collected in agreement with American Society of Clinical Oncology (ASCO) guidelines. The presence and severity of irAEs were graded in agreement with the CTCAE version 4 [26]. Biochemical markers were the neutrophil on lymphocyte ratio (N/L) on the day of ICB initiation or, if absent, the most recent in the week before. The highest C reactive protein (CRP) and LDH levels were collected during the precedent hospitalization or during ICI initiation (if done in an inpatient setting), and the lowest albumin level was collected in the same setting.

### 2.5. Statistical Analysis

All statistical analyses were conducted using IBM SPSS Statistics 28.0 and Excel 2016. A *p*-value < 0.05 was considered statistically significant for all analyses. Categorical variables were presented as frequencies and proportions. Continuous variables are presented as mean and standard deviation if their distribution is considered normal. Continuous variables with a non-normal distribution are presented as median and interquartile range. Median PFS and OS were presented along with 95% confidence intervals and Kaplan–Meier plots. Univariate Cox regression was used to calculate hazard ratio (HR) along with confidence intervals for each chosen factor believed to predict PFS and/or OS. ECOG, the number of cycles, and the number of treatment lines were considered categorical variables with the smallest value as the reference. Kaplan–Meier plots were also presented for some categorical variables.

## 3. Results

### 3.1. Patient Characteristics

A total of 49 patients were included in the study. Table 1 provides a demographic overview of the study population. The majority of patients (57.1%; 28/49) were males. Patients’ median age was 64 ± 10 (Table 1). Lung cancer was most frequent (65.3%), followed by melanoma (14.3%) and renal cell carcinoma (RCC) (10.2%). The vast majority of patients (85.7%, 42/49) were with stage IV cancer, while stage III cancer accounted for 14.3% (7/49) of the cases. Of patients included in the analysis, 8.2% (4/49) were ECOG 0, 34.7% (17/49) were ECOG 1, 28.6% (14/49) were ECOG 2, 18.4% (9/49) were ECOG 3 and 2% (1/49) was ECOG 4 (Table 1). We did not find the performance status of 8.2% (4/49) of the study population. Of all the patients, the majority had a BMI in the 18.5 to 24.99 range (46.9%, 23/49) (Table 1), and 24.5% (12/49) had more than 10% weight loss within 6 months. We did not find weight measurements for 51% (25/49) of the study population.

### 3.2. Treatment Characteristics

**Figure 1:**
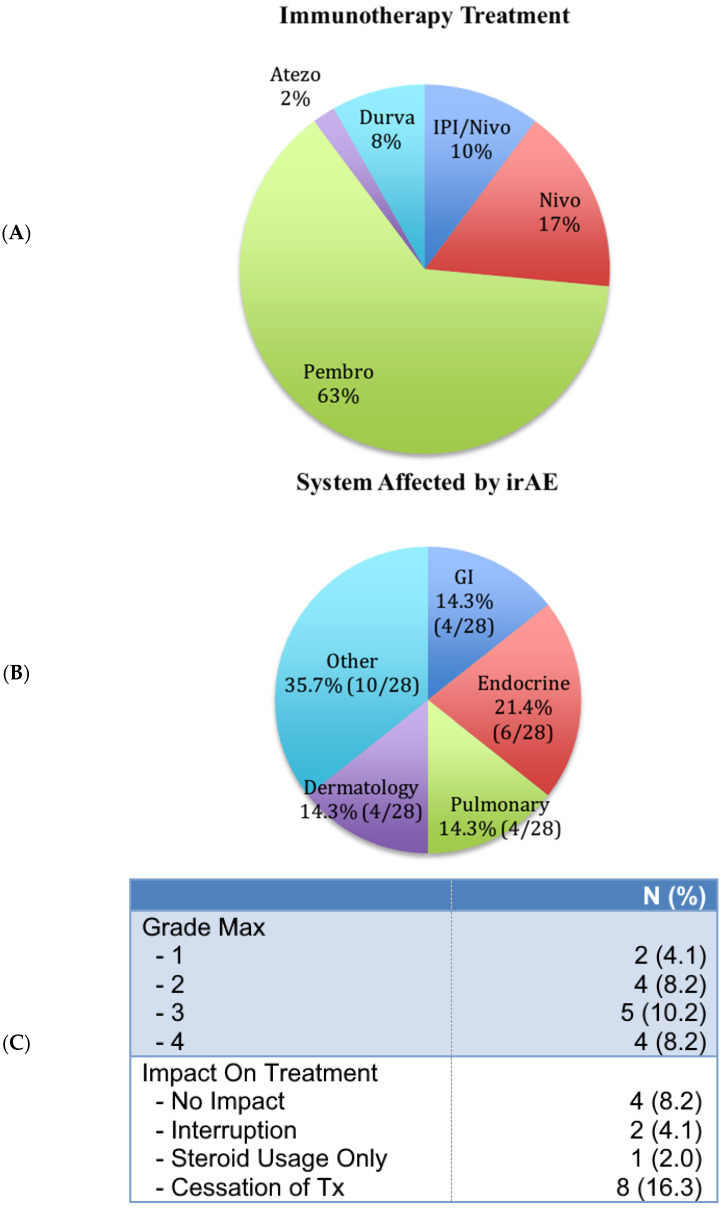
Characterization of immunotherapy treatment. (**A**) Type of immunotherapy treatment. (**B**) Systems affected by irAE. (**C**) Maximum grade of irAE experienced by patients.

Of all the patients included in the study, 67.3% (33/49) were naïve to any prior systemic therapy, while 32.7% (16/49) received at least one line of prior systemic therapy (Table 2). Also, 40.8% (20/49) received prior curative intent radiotherapy, while 59.2% (29/49) were radiotherapy naïve. As for other treatments, 53.1% (26/49) had surgical resection of either primary tumor or metastases. Combination ICIs, namely Ipilimumab/Nivolumab (IPI/Nivo), have been used to treat 10.2% (5/49) of the patients. The rest (88.8%) received ICI monotherapy. The most frequent ICI monotherapy was Pembrolizumab (63.3%, n = 33), followed by Nivolumab (16.3%, n = 8), Durvalumab (8.2%, n = 4), and Atezolizumab (2.0%, n = 1) (Figure 1A).

### 3.3. ICI-Related Adverse Events

All grades compounded, 28 irAE were reported in 30.6% (15/49) (Figure 1B) of patients. The most frequent irAE, 21.4% (6/28), involved the endocrine system (hypothyroidism, adrenal insufficiency, or pancreatic insufficiency) (Figure 1C). Colitis, pneumonitis, and dermatitis accounted for 14.3% (4/28) of reported irAEs each (Figure 1C). Grade three irAE were more common with 10.2% (5/49) frequency (Figure 1B). Grade three and four irAEs combined accounted for the majority of patients who experienced irAE, with a total of 19.4% (9/49) (Figure 1B). Only 4.1% (2/49) experienced grade one, and 8.2% (4/49) experienced grade two irAE (Figure 1B). Treatment was interrupted in 4.1% (2/49) and ceased in 16.3% (8/49) (Figure 1C). Corticosteroid alone was used in only 2% (1/49) for the treatment of irAE, whilst 8.2% (4/49) had irAE without any impact on their ICI treatment (Figure 1C).

### 3.4. Effectiveness of Immunotherapy

Median overall survival (OS) was 3.22 months (95% CI [1.60–4.84]). Median progression-free survival (PFS) was 2.76 months (95% CI [2.17–3.35]) (Table 3 and Table 4, Supplemental Figure S1). Objective response rate (ORR) was 38.9% (7/18) for this cohort of patients receiving ICI treatment. Patients who received at least six cycles of immunotherapy had better overall survival (HR 0.040 [0.009–0.186]; *p* < 0.001) and progression-free survival (HR 0.123 [0.039–0.384]; *p* < 0.001) compared to patients who received one to three cycles (Table 3 and Table 4, Supplemental Figure S1). Patients who received four to six cycles of immunotherapy also had better overall survival (HR 0.335 [0.152–0.738]; *p* < 0.007) and progression-free survival (HR 0.477 [0.193–1.178]; *p* = 0.109) compared to patients who received one to three cycles (Table 3 and Table 4, Supplemental Figure S1). Patients with poor performance status, defined as ECOG 3 or 4, had worse overall survival (HR 5.666 [1.207–26.594]; *p =* 0.028) and progression-free survival (HR 4.136 [0.867–19.733]; *p* = 0.075) on immunotherapy compared to patients with ECOG 0 (Table 3 and Table 4, Supplemental Figure S1). Patients treated with immunotherapy in the first line, compared to the second or latter lines, had a better overall survival (HR 2.603 [1.174–5.769]; *p* = 0.019) and a trend toward a better progression-free survival (HR 2.486 [0.719–8.595]; *p* = 0.150). Low levels of serum albumin (HR 0.917 [0.852–0.987]; *p* = 0.021) and elevated levels of serum LDH (HR 2.224 [1.469–3.367]; *p* < 0.001) were associated with a reduced overall survival (Table 3). However, neither serum albumin nor serum LDH levels showed a statistically significant association with progression-free survival (Table 4). An elevated neutrophil to lymphocyte ratio was associated with a reduced progression-free survival (HR 1.070) [1.014–1.128]; *p* = 0.013) but showed no statistically significant association with overall survival (HR 1.035 [0.985–1.088]; *p* = 0.168) (Table 3 and Table 4). Significant weight loss, defined as at least 10% weight loss within 6 months prior to treatment, showed a trend toward reduced overall survival (HR 2.007; *p* = 0.136) and progression-free survival (HR 2.849; *p* = 0.085) (Table 3 and Table 4). Serum CRP did not show any association with overall survival or progression-free survival (Table 3 and Table 4).

## 4. Discussion

In this single-center retrospective study, we explored the effectiveness of ICIs in frail hospitalized patients or in patients up to 3 months following hospitalization. Our findings reveal that OS (3.2 months) and PFS (2.8 months) were significantly reduced in this fragile population compared to many phase three randomized controlled trials where ICIs have proven to be effective in many solid cancer types. In some cancer types, like melanoma, RCC, and non-small cell lung cancer (NSCLC), up to 30–50% sustained long-term response to ICIs have been reported [8,27]. However, these trials did not include fragile patients with poor performance status (ECOG ≥ 2), with significant co-morbidity, or hospitalized patients. Some fragile patients do respond to ICIs, as evidenced by many case reports and case series [14,28]. Yet, the ratio of patients with successful treatment to futile treatment or factors associated with response to ICIs in fragile patients remains unknown.

We have identified 49 patients who received ICIs while being hospitalized or within 3 months after hospitalization. The fragility of this cohort of patients was highlighted by the median age of 64 years, roughly half of the patients (49.0% with ECOG ≥ 2) had poor performance status, and the majority of the patients (85.7% with Stage IV disease) had an advanced stage cancer (Table 1). The advanced nature of the disease is reflected by the fact that the majority of the patients had prior localized treatment (53.1% surgery and 40.8% radiation) (Table 2). Lung cancer was most frequent in our cohort of patients (65.3%—Table 1), perhaps due to the highly prevalent nature of this disease and high sensitivity to ICIs [29,30]. Two other cancers that are highly sensitive to ICIs, namely melanoma and RCC, were second (14.3%) and third (10.2%) most frequent in our cohort of patients (Table 3) [31]. Pembrolizumab has been approved as monotherapy for stage IV NSCLC with a combined positive score (CPS) ≥ 50 or in combination with platinum doublet with CPS ≥ 1 [29,30]. As most patients in our cohort had lung cancer, it is not surprising that Pembrolizumab was the most frequent ICI.

While irAEs affecting the endocrine system (12.2% (6/49)), gastrointestinal system (8,2% (4/49)), and skin (8.2% 4/49)) were comparable, pulmonary toxicity (8.2% (4/49)) almost doubled what is reported in the literature (Figure 1) [32]. This discrepancy is perhaps explained by the fact that the vast majority of our patients (65.3%—Table 1) already had a vulnerable pulmonary system due to their primary malignancy. All grades combined immune-related adverse events (irAEs) were comparable (30.7%) (Figure 1) to what has been described in the literature [32,33]. Many irAEs resulted in either treatment interruption (4.1%) or cessation (16.3%) (Figure 1).

Despite comparable levels of irAEs and severity of adverse events, ORR (38.9%), OS (3.22 months (95% CI [1.60–4.84])) and PFS (2.76 months (95% CI [2.17–3.35]) of ICIs in our cohort of patients were much lower than what has been reported in existing studies [8,29,30]. This may be due to the fragility and perhaps weaker immune system of our patient population. Given that irAEs limit the quality of life of many patients, it is important to identify clinical and laboratory factors associated with response to ICIs. To this extent, low neutrophil to lymphocyte ratio (ratio N/L) has been documented to be a predictive biomarker of good prognosis in melanoma patients [34]. In our analysis, the ratio N/L (Table 3 and Table 4) shows a statistically significant association with PFS but not with OS. This discrepancy may be due to the small sample size or inherent reflection of the ratio N/L as a poor predictor of prognosis in fragile patients. On the other hand, serum LDH and albumin levels (Table 3 and Table 4) were found to have a statistically significant association with OS but not with PFS. This observation, especially serum LDH level association with prognosis, is consistent with existing studies [35,36,37].

Performance status (ECOG) remains a widely used determinant in assessing fitness to receive systemic therapies, including ICIs [19]. In concordance with the approach, ECOG ≥ 3 was associated with significantly worse PFS and OS in fragile patients (Table 3 and Table 4). Unlike chemotherapy, which kills cancer cells, immunotherapy potentiates the immune system to attack cancer cells. Consequently, it generally takes three to four cycles of ICIs before starting to see a response to treatment [20]. In line with that argument, patients who received four to six cycles of ICIs had a significantly better OS (Table 3) and a trend toward better PFS (Table 4). Patients who had six or more cycles of ICIs had significantly improved OS and PFS (Table 3 and Table 4). Overall, there was a trend toward better treatment response to ICIs in early lines or systemic treatment in naïve patients (Table 3 and Table 4). There was also a trend toward statistically significant poor response observed in terms of OS and PFS in patients losing more than 10% weight in 6 months prior to receiving ICIs (Table 3 and Table 4). This is in line with the observation that patients who lose significant weight have an advanced disease due to the catabolic nature of cancer. As such, perhaps a statistical significance could have been observed, had the sample size been bigger.

## 5. Conclusions

Although ICIs are an effective treatment against many solid cancers, irAEs are common and adversely affect the quality of life of many patients. Sometimes, severe irAEs could even lead to death. In hospitalized or recently hospitalized (≤3 months) patients, ICIs were far less effective, as evidenced by a lower ORR, PFS, and OS. However, certain clinical factors like good performance status (ECOG ≤ 2) and capacity to receive at least three treatments of ICIs, as well as normal levels of serum albumin, and LDH could be useful in selecting patients suitable to receive ICIs. Given the retrospective nature of the analysis, work from other centers is needed to consolidate the findings from this study. Also, a larger sample size may be useful to discern any potential association between significant weight loss (≥10% weight loss in 6 months) and the effectiveness of ICIs in fragile patient populations.

## Figures and Tables

**Table 1:** Characteristics of the study patient population. BMI (body mass index), RCC (renal cell carcinoma), HNSCC (head and neck squamous cell carcinoma), CRC (colorectal cancer), NA (Not available).

Characteristics	N (%)
Sex	
- Female	21 (42.9)
- Male	28 (57.1)
Age	
- Median	64 (37–85)
- Mean	64.4 ± 10
- Range	37–85
Cancer type	
- Lung	32 (65.3)
- Melanoma	7 (14.3)
- RCC	5 (10.2)
- HNSCC	2 (4.1)
- CRC	1 (2.0)
- Other	2 (4.1)
Stage	
- 3	7 (14.3)
- 4	42 (85.7)
ECOG	
- 0	4 (8.2)
- 1	17 (34.7)
- 2	14 (28.6)
- 3	9 (18.4)
- 4	1 (2.0)
- NA	4 (8.2)
BMI	
- <18.5	3 (6.1)
- 18.5–24.9	23 (46.9)
- 25–29.9	14 (28.6)
- 30–34.9	8 (16.3)
- 35–39.9	0 (0)
- >40	1 (2.0)
Weight loss (≥10% in 6 months)	
- Yes	12 (24.5)
- No	12 (24.5)
- Unknown	25 (51)

**Table 2:** Characterization of prior treatment history of the study population. Radiotherapy varied from 30 to 100 Gy. Surgery includes resection of primary tumor or metastatectomy.

Prior Treatment	N (%)
Chemotherapy	
- Yes	16 (32.7)
- No	33 (67.3)
Radiation	
- Yes	20 (40.8)
- No	29 (59.2)
Surgery	
- Yes	26 (53.1)
- No	23 (46.9)

**Table 3:** Overall survival outcome of patients treated with immunotherapy during hospitalization or within 3 months of hospitalization based on laboratory and clinical variables.

Variable	HR [IC 95%]	*p*-Value
Ratio N/L	1.035 [0.985–1.088]	0.168
CRP (mg/L)	1.002 [0.998–1.007]	0.302
Albumin (g/L)	0.917 [0.852–0.987]	0.021
LDH (U/L)	2.224 [1.469–3.367]	<0.001
ECOG		
0	Ref.	-
1	1.057 [0.231–4.836]	0.943
2	3.766 [0.845–16.787]	0.082
3–4	5.666 [1.207–26.594]	0.028
Number of cycles		
1–3	Ref.	-
4–6	0.335 [0.152–0.738]	0.007
>6	0.040 [0.009–0.186]	<0.001
Line of treatment		
1	Ref.	-
≥2	2.603 [1.174–5.769]	0.019
Weight loss (≥10% in 6 months)	2.007 [0.803–5.014]	0.136

**Table 4:** Progression-free survival outcome of patients treated with immunotherapy during hospitalization or within 3 months of hospitalization based on laboratory and clinical variables.

Variable	HR [IC 95%]	*p*-Value
Ratio N/L	1.070 [1.014–1.128]	0.013
CRP (mg/L)	1.002 [0.996–1.009]	0.442
Albumin (g/L)	0.971 [0.872–1.082]	0.598
LDH (U/L)	19.128 [0.031–11674.733]	0.367
ECOG		
0	Ref.	-
1	0.833 [0.230–3.014]	0.781
2	1.391 [0.307–6.300]	0.668
3–4	4.136 [0.867–19.733]	0.075
Number of cycles		
1–3	Ref.	-
4–6	0.477 [0.193–1.178]	0.109
>6	0.123 [0.039–0.384]	<0.001
Line of treatment		
1	Ref.	-
≥2	2.486 [0.719–8.595]	0.150
Weight loss (≥10% within 6 months)	2.849 [0.865–9.382]	0.085

## Data Availability

The original contributions presented in the study are included in the article/Supplementary Material. Further inquiries can be directed to the corresponding author.

## Supplementary Materials

The following supporting information can be downloaded at: https://www.mdpi.com/article/10.3390/curroncol31110518/s1, Figure S1: Survival outcome analysis based on clinical variables.

## References

[1] Sung H., Ferlay J., Siegel R.L., Laversanne M., Soerjomataram I., Jemal A., Bray F. (2021). Global Cancer Statistics 2020: GLOBOCAN Estimates of Incidence and Mortality Worldwide for 36 Cancers in 185 Countries. CA Cancer J. Clin..

[2] Rizzo A., Mollica V., Santoni M., Massari F. (2021). Cancer Immunotherapy: Current and Future Perspectives on a Therapeutic Revolution. J. Clin. Med..

[3] Korman A.J., Garrett-Thomson S.C., Lonberg N. (2022). The foundations of immune checkpoint blockade and the ipilimumab approval decennial. Nat. Rev. Drug Discov..

[4] Scott E.C., Baines A.C., Gong Y., Moore R., Pamuk G.E., Saber H., Subedee A., Thompson M.D., Xiao W., Pazdur R. (2023). Trends in the approval of cancer therapies by the FDA in the twenty-first century. Nat. Rev. Drug Discov..

[5] Hodi F.S., O’Day S.J., McDermott D.F., Weber R.W., Sosman J.A., Haanen J.B., Gonzalez R., Robert C., Schadendorf D., Hassel J.C. (2010). Improved survival with ipilimumab in patients with metastatic melanoma. N. Engl. J. Med..

[6] Iranzo P., Callejo A., Assaf J.D., Molina G., Lopez D.E., Garcia-Illescas D., Pardo N., Navarro A., Martinez-Marti A., Cedres S. (2022). Overview of Checkpoint Inhibitors Mechanism of Action: Role of Immune-Related Adverse Events and Their Treatment on Progression of Underlying Cancer. Front. Med..

[7] Waldmann T.A. (2003). Immunotherapy: Past, present and future. Nat. Med..

[8] Hodi F.S., Chiarion-Sileni V., Gonzalez R., Grob J.J., Rutkowski P., Cowey C.L., Lao C.D., Schadendorf D., Wagstaff J., Dummer R. (2018). Nivolumab plus ipilimumab or nivolumab alone versus ipilimumab alone in advanced melanoma (CheckMate 067): 4-year outcomes of a multicentre, randomized, phase 3 trial. Lancet Oncol..

[9] Toribio-Vazquez C., Gomez Rivas J., Yebes A., Carrion D.M., Quesada-Olarte J., Trelles C.R., Alvarez-Maestro M., van der Poel H., Martinez-Pineiro L. (2020). Immunotherapy toxicity. Diagnosis and treatment. Arch. Esp. Urol..

[10] Darnell E.P., Mooradian M.J., Baruch E.N., Yilmaz M., Reynolds K.L. (2020). Immune-Related Adverse Events (irAEs): Diagnosis, Management, and Clinical Pearls. Curr. Oncol. Rep..

[11] Sosa A., Lopez Cadena E., Simon Olive C., Karachaliou N., Rosell R. (2018). Clinical assessment of immune-related adverse events. Ther. Adv. Med. Oncol..

[12] Pierro M., Baldini C., Auclin E., Vincent H., Varga A., Martin Romano P., Vuagnat P., Besse B., Planchard D., Hollebecque A. (2022). Predicting Immunotherapy Outcomes in Older Patients with Solid Tumors Using the LIPI Score. Cancers.

[13] Muchnik E., Loh K.P., Strawderman M., Magnuson A., Mohile S.G., Estrah V., Maggiore R.J. (2019). Immune Checkpoint Inhibitors in Real-World Treatment of Older Adults with Non-Small Cell Lung Cancer. J. Am. Geriatr. Soc..

[14] Nie N.F., Liu Z.L., Feng M.X., Liu L., Luo N., Li L., He Y. (2021). Lazarus type response to immunotherapy in three patients with poor performance status and locally advanced NSCLC: A case series and literature review. Ann. Palliat. Med..

[15] Magee D.E., Hird A.E., Klaassen Z., Sridhar S.S., Nam R.K., Wallis C.J.D., Kulkarni G.S. (2020). Adverse event profile for immunotherapy agents compared with chemotherapy in solid organ tumors: A systematic review and meta-analysis of randomized clinical trials. Ann. Oncol..

[16] Tsimberidou A.M., Fountzilas E., Nikanjam M., Kurzrock R. (2020). Review of precision cancer medicine: Evolution of the treatment paradigm. Cancer Treat. Rev..

[17] Pietrantonio F., Loupakis F., Randon G., Raimondi A., Salati M., Trapani D., Pagani F., Depetris I., Maddalena G., Morano F. (2020). Efficacy and Safety of Immune Checkpoint Inhibitors in Patients with Microsatellite Instability-High End-Stage Cancers and Poor Performance Status Related to High Disease Burden. Oncologist.

[18] Blum S.M., Rouhani S.J., Sullivan R.J. (2023). Effects of immune-related adverse events (irAEs) and their treatment on antitumor immune responses. Immunol. Rev..

[19] Ao G., de Miguel M., Gomes A., Liu R., Boni V., Moreno I., Cardenas J.M., Cubillo A., Ugidos L., Calvo E. (2021). Toxicity and antitumor activity of novel agents in elderly patients with cancer included in phase 1 studies. Investig. New Drugs.

[20] Marron T.U., Ryan A.E., Reddy S.M., Kaczanowska S., Younis R.H., Thakkar D., Zhang J., Bartkowiak T., Howard R., Anderson K.G. (2021). Considerations for treatment duration in responders to immune checkpoint inhibitors. J. Immunother. Cancer.

[21] Brands X., Haak B.W., Klarenbeek A.M., Otto N.A., Faber D.R., Lutter R., Scicluna B.P., Wiersinga W.J., van der Poll T. (2020). Concurrent Immune Suppression and Hyperinflammation in Patients With Community-Acquired Pneumonia. Front. Immunol..

[22] Yende S., Kellum J.A., Talisa V.B., Peck Palmer O.M., Chang C.H., Filbin M.R., Shapiro N.I., Hou P.C., Venkat A., LoVecchio F. (2019). Long-term Host Immune Response Trajectories Among Hospitalized Patients with Sepsis. JAMA Netw. Open.

[23] Yende S., D’Angelo G., Kellum J.A., Weissfeld L., Fine J., Welch R.D., Kong L., Carter M., Angus D.C., Gen I.M.S.I. (2008). Inflammatory markers at hospital discharge predict subsequent mortality after pneumonia and sepsis. Am. J. Respir. Crit. Care Med..

[24] Seymour L., Bogaerts J., Perrone A., Ford R., Schwartz L.H., Mandrekar S., Lin N.U., Litiere S., Dancey J., Chen A. (2017). iRECIST: Guidelines for response criteria for use in trials testing immunotherapeutics. Lancet Oncol..

[25] Azam F., Latif M.F., Farooq A., Tirmazy S.H., AlShahrani S., Bashir S., Bukhari N. (2019). Performance Status Assessment by Using ECOG (Eastern Cooperative Oncology Group) Score for Cancer Patients by Oncology Healthcare Professionals. Case Rep. Oncol..

[26] National Cancer Institute (U.S.) (2009). Common Terminology Criteria for Adverse Events (CTCAE).

[27] Nasser N.J., Gorenberg M., Agbarya A. (2020). First line Immunotherapy for Non-Small Cell Lung Cancer. Pharmaceuticals.

[28] Grivas P., Plimack E.R., Balar A.V., Castellano D., O’Donnell P.H., Bellmunt J., Powles T., Hahn N.M., de Wit R., Bajorin D.F. (2020). Pembrolizumab as First-line Therapy in Cisplatin-ineligible Advanced Urothelial Cancer (KEYNOTE-052): Outcomes in Older Patients by Age and Performance Status. Eur. Urol. Oncol..

[29] Reck M., Rodriguez-Abreu D., Robinson A.G., Hui R., Csoszi T., Fulop A., Gottfried M., Peled N., Tafreshi A., Cuffe S. (2016). Pembrolizumab versus Chemotherapy for PD-L1-Positive Non-Small-Cell Lung Cancer. N. Engl. J. Med..

[30] Mok T.S.K., Wu Y.L., Kudaba I., Kowalski D.M., Cho B.C., Turna H.Z., Castro G., Srimuninnimit V., Laktionov K.K., Bondarenko I. (2019). Pembrolizumab versus chemotherapy for previously untreated, PD-L1-expressing, locally advanced or metastatic non-small-cell lung cancer (KEYNOTE-042): A randomised, open-label, controlled, phase 3 trial. Lancet.

[31] Mazza C., Escudier B., Albiges L. (2017). Nivolumab in renal cell carcinoma: Latest evidence and clinical potential. Ther. Adv. Med. Oncol..

[32] Sacchi de Camargo Correia G., Pai T., Li S., Connor D., Zhao Y., Lou Y., Manochakian R. (2023). Immune-Related Adverse Events in Patients with Lung Cancer. Curr. Oncol. Rep..

[33] Fu Y., Zheng Y., Wang P.P., Ding Z.Y. (2021). Toxicities of Immunotherapy for Small Cell Lung Cancer. Front. Oncol..

[34] Li Y., Meng Y., Sun H., Ye L., Zeng F., Chen X., Deng G. (2022). The Prognostic Significance of Baseline Neutrophil-to-Lymphocyte Ratio in Melanoma Patients Receiving Immunotherapy. J. Immunother..

[35] Popovic A., Petkovic I., Dimitrijevic A., Jovic A. (2023). Prognostic Value of Lactate Dehydrogenase in Patients with Melanoma Treated with Pembrolizumab. Acta Dermatovenerol. Croat.

[36] Wei Y., Xu J., Huang X., Xie S., Lin P., Wang C., Guo Y., Zou S., Zhao Z., Wen W. (2023). C-reactive protein and lactate dehydrogenase serum levels potentially predict the response to checkpoint inhibitors in patients with advanced non-small cell lung cancer. J. Thorac. Dis..

[37] Shen J., Chen Z., Zhuang Q., Fan M., Ding T., Lu H., He X. (2016). Prognostic Value of Serum Lactate Dehydrogenase in Renal Cell Carcinoma: A Systematic Review and Meta-Analysis. PLoS ONE.

