# Supplementary figures and images for: Evaluating the Efficacy of Immunotherapy in Fragile Hospitalized Patients
Source: Curr Oncol. 2024 Nov 10;31(11):7040–50. doi: 10.3390/curroncol31110518 (PMC11593166; doi:10.3390/curroncol31110518)

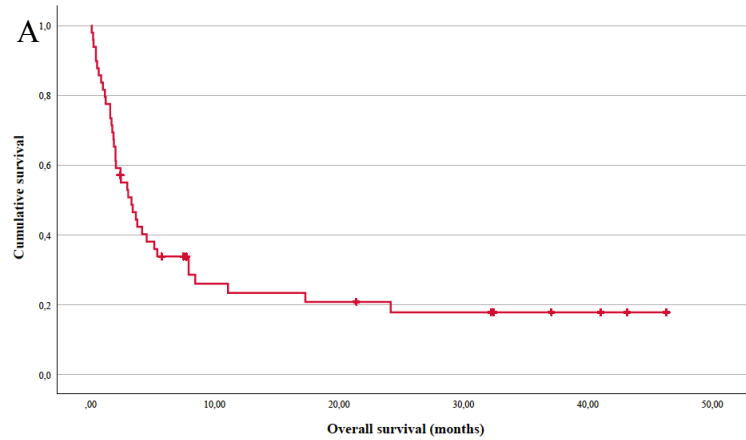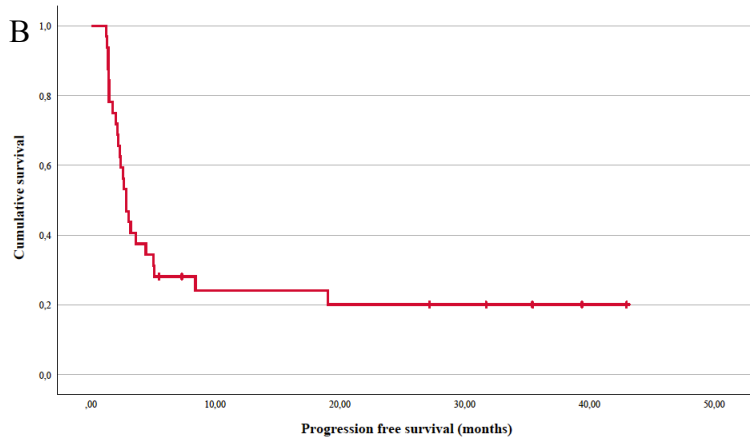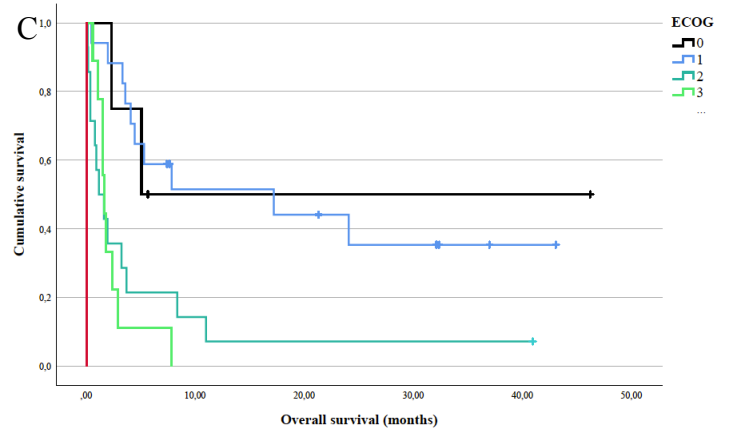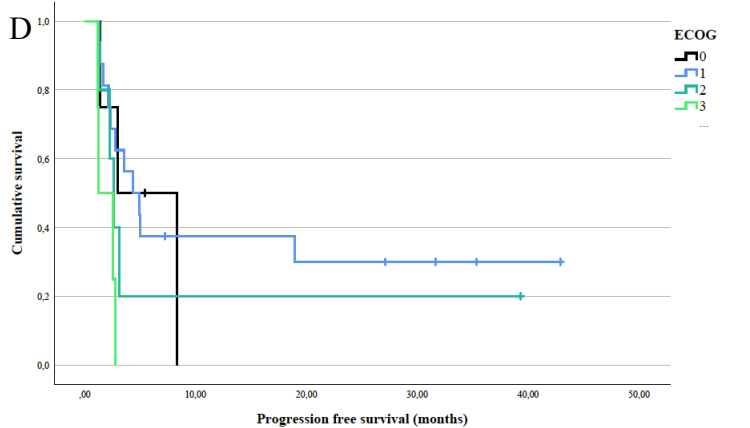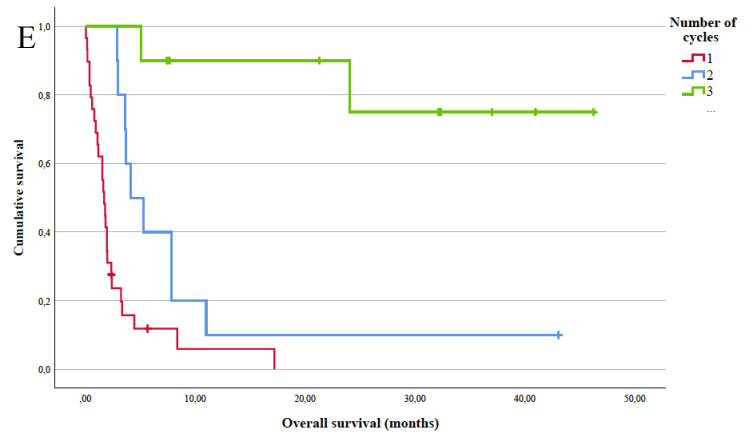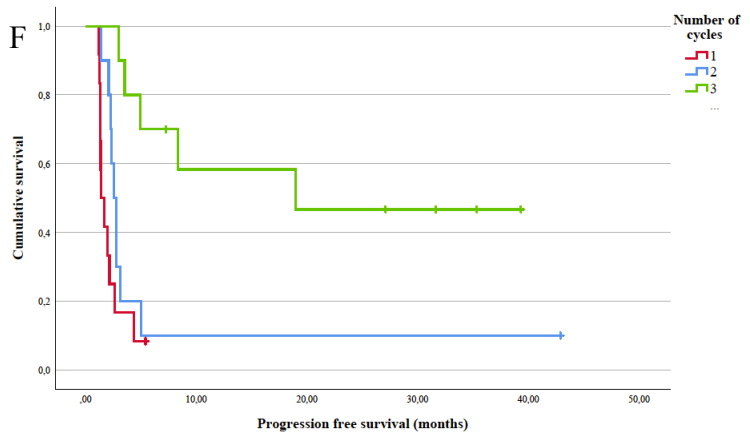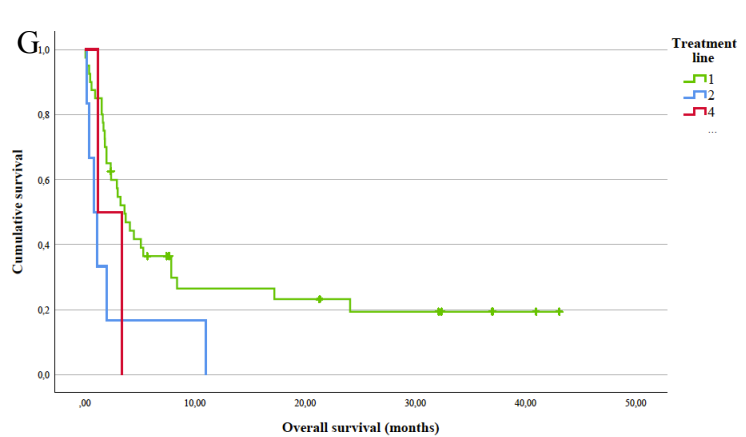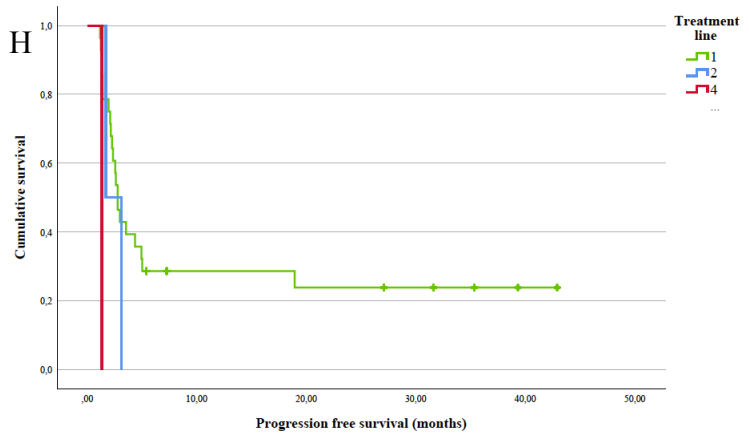

Supplement: Supplementary file 1 [file curroncol-31-00518-s001.zip › curroncol-3244887-Figure S1.pdf]
